# Determinants of Serum- and Plasma Sphingosine-1-Phosphate Concentrations in a Healthy Study Group

**DOI:** 10.1055/s-0040-1701205

**Published:** 2020-01-23

**Authors:** Günter Daum, Martin Winkler, Eileen Moritz, Tina Müller, Maria Geffken, Mirjam von Lucadou, Munif Haddad, Sven Peine, Rainer H. Böger, Axel Larena-Avellaneda, Eike Sebastian Debus, Markus Gräler, Edzard Schwedhelm

**Affiliations:** 1 Clinic and Polyclinic for Vascular Medicine, University Heart and Vascular Center, Hamburg, Germany; 2 German Center for Cardiovascular Research (DZHK), Partner Site Hamburg/Kiel/Lübeck (GD, ES, MvL) and Greifswald (EM), Berlin, Germany; 3 Department of Anesthesiology, University Medical Center Hamburg-Eppendorf, Hamburg, Germany; 4 Department of Anesthesiology and Intensive Care Medicine, University Medicine, Göttingen, Germany; 5 Institute for Clinical Pharmacology and Toxicology, University Medical Center Hamburg-Eppendorf, Hamburg, Germany; 6 Institute of Pharmacology, Department of General Pharmacology, University Medicine, Greifswald, Germany; 7 Department of Anesthesiology and Intensive Care Medicine, Center for Sepsis Control and Care (CSCC), and the Center for Molecular Biomedicine (CMB), Jena University Hospital, Jena, Germany; 8 Institute for Transfusion Medicine, University Medical Center Hamburg, Eppendorf, Germany; 9 Institute for Clinical Chemistry, University Medical Center Hamburg, Eppendorf, Germany

**Keywords:** sphingosine-1-phosphate, lipoproteins, blood

## Abstract

**Introduction** To correctly interpret plasma- or serum-sphingosine-1-phosphate (S1P) concentrations measured in clinical studies it is critical to understand all major determinants in healthy controls.

**Methods** Serum- and plasma-S1P from 174 healthy blood donors was measured by liquid chromatography-tandem mass spectrometry and correlated to clinical laboratory data. Selected plasma samples, 10 with high and 10 with low S1P concentrations, were fractionated into very low-density lipoprotein (VLDL)-, low density lipoprotein (LDL)-, high density lipoprotein (HDL)-, and lipoprotein-free fractions. S1P was then measured in each fraction to determine its distribution.

**Results** The mean S1P concentration in serum (1.04 ± 0.24 nmol/mL) was found 39% higher compared with plasma (0.75 ± 0.16 nmol/mL) and overall was not different between men and women. Only when stratified for age and gender, older women were found to exhibit higher circulatory S1P levels than men. In plasma, S1P levels correlate to red blood cell (RBC) counts but not to platelet counts. Conversely, serum-S1P correlates to platelet counts but not to RBC counts. In addition, eosinophil counts are strongly associated with serum-S1P concentrations. Both serum- and plasma-S1P correlate to total cholesterol but not to HDL-C. The distribution of S1P between VLDL-, LDL-, HDL-, and lipoprotein-free fractions is independent of total plasma-S1P concentrations. S1P concentrations in HDL but not in LDL are highly variable.

**Conclusion** These data indicate S1P concentrations in plasma and serum to be differentially associated with cell counts and S1P carrier proteins. Besides platelets, eosinophil counts are identified as a novel determinant for serum-S1P concentrations further suggesting a role for S1P in eosinophil pathologies.

## Introduction

Sphingosine-1-phosphate (S1P) is a bioactive lipid regulating a plethora of physiological as well as pathophysiological processes via binding to 5 specific G protein-coupled receptors (S1PR1-5). 1 2 3 S1P is present at high concentrations in blood where it is mainly bound to high density lipoprotein (HDL) and albumin. 4 In the vasculature, S1P is critical to maintain the endothelial barrier function via S1PR1 signaling. 5 6 7 The S1P/S1PR1 axis is also required for lymphocytes to detect an S1P gradient (high in blood, low in tissue) which they utilize to egress from lymphatic organs. 8 9 These properties of S1P gave rise to several preclinical studies testing for associations between circulatory S1P concentrations and clinical parameters in Dengue fever, 10 11 sepsis, 7 12 13 and atherosclerotic diseases. 14 15 16 In these studies, S1P has been measured in either serum or plasma whereby higher concentrations are generally seen in serum. This difference is attributed to platelets that release S1P during coagulation. 17 18 As of today, two S1P transporters have been identified in platelets, the ATP-dependent multidrug resistance protein 4 (Mrp4) 19 and the major facilitator superfamily transporter 2b (Mfsd2b). 20 Interpretations of S1P measurements in clinical settings, however, are hampered not only by the high variability of circulating S1P concentrations found in humans, with a reference interval for serum-S1P between 0.53 and 1.24 nmol/mL, 21 but also by seemingly contradictory observations made depending on whether serum-S1P or plasma-S1P was measured. 14 15 It is therefore important to identify major determinants of S1P concentrations in serum and plasma in a healthy study group to define a reliable reference for future clinical studies.

## Materials and Methods

### Study Group

All blood donations were performed at the Institute for Transfusion Medicine at the University Medical Center Hamburg-Eppendorf in accordance with the latest guidelines of the German Federal Medical Council (Bundesärztekammer) issued in 2010 that specifically exclude blood donations from subjects with severe health problems including clinically relevant cardiovascular diseases. In our group of 174 blood donors, only residual amounts of anonymous blood samples have been used which are routinely taken from all blood donors and would have been discarded otherwise. All blood donors gave their general written consent to the use of their blood samples for scientific studies in an anonymized form. The anonymous use of this material is in compliance with a vote by the Ethics Committee of the German Medical Association.

### Laboratory Measurements

All measurements of blood parameters (blood groups (O+/−, A+/−, B+/−, and AB+/−), cell counts (red blood cells [RBCs], total leukocytes, platelets, lymphocytes, monocyte, neutrophils, eosinophils, and basophils), albumin, total cholesterol, HDL-cholesterol (HDL-C), low density lipoprotein cholesterol (LDL-C) and triglycerides were performed by standardized assays in the Institute for Clinical Chemistry and the Institute for Transfusion Medicine, both at the University Medical Center Hamburg-Eppendorf, Germany.

### Lipoprotein Fractionation

Lipoproteins were sequentially precipitated by adding increasing concentrations of Na _3_ P(W _3_ O _10_ ) _4_ as previously described. 22 All procedures were performed at room temperature. For chylomicron and very low-density lipoprotein (VLDL) precipitation, 25 μL of a 1% Na _3_ P(W _3_ O _10_ ) _4_ and 25 μL of a 2 M MgCl _2_ solution were added to 500-μL plasma. After brief mixing and 15-minutes incubation, the samples were centrifuged for 10 minutes at 6,000 rcf, and supernatants were transferred to new tubes for further precipitation. For LDL precipitation, 25 μL of a 4% Na _3_ P(W _3_ O _10_ ) _4_ solution were added to the first supernatant. After 15-minutes incubation, the samples were centrifuged for 10 minutes at 6,000 rcf, and the supernatants were again transferred to new tubes. For HDL precipitation, 25 μL of a 40% Na _3_ P(W _3_ O _10_ ) _4_ and 25 μL of a 2M MgCl _2_ solution were added to the second supernatant. After 2-hour incubation, the samples were centrifuged for 30 minutes at 20,000 rcf. The supernatant was the lipoprotein-free (albumin) fraction. If not used for S1P measurements immediately, fractions were stored at −20°C.

### S1P Measurements

After venous puncture, blood samples (heparin plasma and serum) were centrifuged for 10 minutes and supernatants were stored at −80°C until use. S1P measurements were conducted at two sites, both using liquid chromatography coupled to tandem mass spectrometry (LC-MS/MS). Serum- and plasma-S1P of all study participants was measured in one batch at the Institute of Clinical Pharmacology and Toxicology, University Medical Center Hamburg-Eppendorf, Germany as previously described. 21 In brief, 20 µL of sample was incubated with 20 µL of the internal standard (1 µM [16,17,18- ^2^ H _7_ ]-S1P; S1P-d _7_ , Avanti Polar Lipids, Alabaster, United States). After protein precipitation with acetonitrile/water, 80/20 (vol/vol), the extracts were subjected to reverse-phase chromatography on a Zorbax SB-C8 column (2.1 × 50 mm; Agilent Technologies, Santa Clara, United States). S1P was eluted with a binary gradient for 6 minutes (methanol/acetonitrile/0.1% formic acid: 2.5/2.5/95 to 30/30/40, vol/vol/vol) and measured by positive electrospray ionization (ESI) tandem mass spectrometry (Varian L1200 MS/MS, Agilent Technologies). Multiple reaction monitoring (MRM) transitions were monitored as follows: the m/z 380 to 264 transition of S1P and the m/z 387 to 271 transition of S1P-d _7_ . The S1P content in lipoprotein fractions and serum-albumin was measured at the Department of Anesthesiology and Intensive Care Medicine, Jena University Hospital, Germany as previously reported 22 : After addition of C17-base S1P as internal standard (100 pmol/sample, Avanti Polar Lipids), samples were transferred to glass centrifuge tubes and adjusted to a final volume of 1 mL with H _2_ O. After addition of 0.3 mL 6 N HCl, 1 mL methanol, and 2 mL chloroform, samples were vigorously vortexed for 10 minutes. Aqueous and chloroform phases were separated by centrifugation for 3 minutes at 1,900 rcf, and the lower chloroform phase was transferred into a new glass centrifuge tube. After a second round of lipid extraction with additional 2-mL chloroform per milliliter aqueous sample, the two chloroform phases were combined and vacuum-dried at 60°C for 50 minutes using a vacuum concentrator. The extracted lipids were dissolved in 100 μL methanol/chloroform (4:1, vol/vol) and stored at −20°C. Liquid chromatographic resolution of all analytes was achieved using a 2 × 60 mm MultoHigh C18 reversed phase column with a 3-μm particle size (CS-Chromatographie Service, Langerwehe, Germany). The column was equilibrated with 10% methanol and 90 of 1% formic acid in H _2_ O for 5 minutes, followed by sample injection and 15-minutes elution with 100% methanol with a flow rate of 300 μL/min. Detection was performed with the QTrap triple-quadrupole mass spectrometer (Sciex, Redwood City, United States) interfaced with the 1100 series chromatograph (Agilent Technologies), the Hitachi Elite LaChrom column oven (VWR, Darmstadt, Germany), and the Spectra System AS3500 autosampler (Thermo Separation Products, Waltham, United States). Positive ESI-LC-MS/MS analysis was used for detection of S1P and C17-S1P. MRM-transitions were as follows: S1P m/z 380/264 and C17-S1P m/z 366/250. For all measurements, calibration curves were generated by measuring increasing concentrations of S1P (0–3 μM).

### Statistical Analyses

Statistical analyses were performed using GraphPad Prism (version 6.07; Graph Pad, La Jolla, United States). The nonparametric Mann–Whitney test was used to test the significance of differences between the two groups, the Kruskal–Wallis analysis of variance (ANOVA) was used for comparing more than two groups. Associations were analyzed by calculating the Spearman's correlation coefficient. *p* < 0.05 is considered significant. Reference intervals were calculated with the Analyze-it software tool according to the Clinical & Laboratory Standards Institute guideline EP28-A3c using quantile regression analysis. More details are given in Table and Figure legends.

## Results

**Fig. 1:**
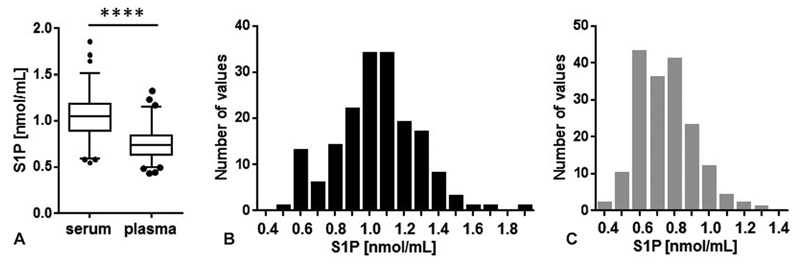
Circulatory S1P concentrations in a blood donor study group. S1P was measured in serum and plasma in a blood donor study group ( *N* = 174) ( **A** ), data are shown as median, interquartile range, and 2.5 to 97.5 percentiles including outliers. **** *p* < 0.0001 (Mann–Whitney test), the frequency distribution for serum-S1P ( **B** , *black bars* ) and plasma-S1P ( **C** , *gray bars* ) is shown. Data for serum but not for plasma passed the D'Agostino & Pearson normality test. S1P, sphingosine-1-phosphate.

**Fig. 2:**
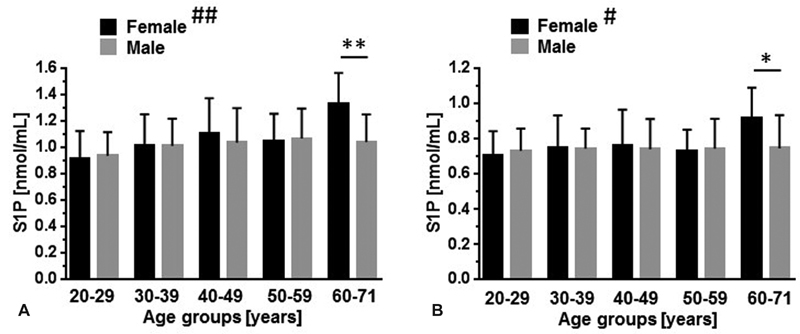
Influence of age and gender on circulatory S1P concentrations. S1P was measured in serum ( **A** ) and plasma ( **B** ) in a blood donor study group ( *N* = 174). Data (mean ± SD) were stratified for age and gender ( *F, female =* *black bars, M, male =* *gray bars* ). 20 to 29 years: *n* = 15 (F), *n* = 16 (M); 30 to 39 years: *n* = 15 (F), *n* = 20 (M); 40 to 49 years: *n* = 10 (F), *n* = 22 (M); 50 to 59 years: *n* = 18 (F). *n* = 31 (M); 60 to 71 years: *n* = 10 (F), *n* = 17 (M). ^#^ *p* < 0.05 (Kruskal–Wallis test, plasma [all female groups]); ^##^ *p* < 0.01 (Kruskal–Wallis test, serum [all female groups]). No significant differences were detected for male groups. * *p* < 0.05, ** *p* < 0.01 (Mann–Whitney test). SD, standard deviation.

S1P concentrations were determined in a study group of healthy blood donors ( *N* = 174) consisting of 106 men and 68 women. The mean S1P concentration in serum (1.04 ± 0.24 nmol/mL) is 39% higher compared with plasma (0.75 ± 0.16 nmol/mL) and does not differ between men and women ( Fig. 1A ; Table 1 ). The lower (2.5%) and upper (97.5%) reference intervals are 0.592 (90% confidence intervals [CI] 0.540–0.614) and 1.509 (90% CI 1.410–1.849) nmol/mL for serum-S1P and 0.490 (90% CI 0.430–0.518) and 1.152 (90% CI 1.031–1.316) nmol/mL for plasma-S1P, respectively. The distribution of serum and plasma S1P values is shown in Fig. 1B,C . In contrast to circulatory S1P concentrations, gender-specific differences are observed for RBC counts, triglycerides, serum-albumin (all higher in men) as well as platelet counts, leukocyte counts, neutrophil counts, and HDL-C (all higher in women; Table 1 ). When the study group is stratified for gender and age, the subgroup of women at the age of 60 or older exhibits higher serum- or plasma-S1P concentrations when compared with an age-matched male group ( Fig. 2 ). To investigate whether major RBC antigens define circulatory S1P concentrations, subgroups were formed according to major blood groups. Although no differences were observed by a Kruskal–Wallis test, serum- and plasma-S1P concentrations were higher for A− compared with B+ groups ( Supplementary Fig. 1 ).

**Table 1:** Baseline characteristics for entire study group and stratified by gender

		All	Female	Male	*p* -Value
Number		174	68	106	–
Age (y)		45.5 (20–71)	44.0 (21–71)	46.4 (20–71)	n.s.
Gender (% male)		61	0	100	–
Serum-S1P	(nmol/mL)	1.04 ± 0.24	1.06 ± 0.26	1.03 ± 0.22	n.s.
Plasma-S1P	(nmol/mL)	0.75 ± 0.16	0.76 ± 0.17	0.74 ± 0.16	n.s.
RBC	(1/pL)	4.84 ± 0.41	4.55 ± 0.28	5.03 ± 0.37	<0.0001
Leukocytes	(1/nL)	6.40 ± 1.62	6.77 ± 1.59	6.16 ± 1.61	<0.05
Platelets	(1/nL)	234 ± 56	257 ± 61	219 ± 48	<0.0001
Lymphocytes	(1/nL)	1.79 ± 0.53	1.83 ± 0.45	1.76 ± 0.57	n.s.
Monocytes	(1/μL)	413 ± 119	397 ± 118	423 ± 120	n.s.
Neutrophils	(1/nL)	3.86 ± 1.30	4.22 ± 1.36	3.63 ± 1.21	<0.01
Eosinophils	(1/μL)	175 ± 84	163 ± 70	182 ± 91	n.s.
Basophils	(1/μL)	100 ± 1	100 ± 0	100 ± 2	n.s.
Total cholesterol	(mg/dL)	186 ± 41	190 ± 45	184 ± 37	n.s.
HDL-C	(mg/dL)	62.7 ± 18.2	70.8 ± 14.7	57.5 ± 18.4	<0.0001
LDL-C	(mg/dL)	98.2 ± 35.0	96.9 ± 33.8	99.0 ± 35.9	n.s.
Triglycerides	(mg/dL)	132 ± 80	120 ± 86	139 ± 76	<0.05
Albumin	(g/L)	43.3 ± 3.0	42.5 ± 3.2	43.8 ± 2.9	<0.05

Abbreviations: HDL-C, high density lipoprotein-cholesterol; LDL-C, low density lipoprotein-cholesterol; n.s., nonsignificant; RBC, red blood cells; S1P, sphingosine-1-phosphate.

Note: Age (mean, min–max), gender, circulatory S1P concentrations (mean ± SD), and clinical laboratory data (mean ± SD) are shown for all study participants as well as for females and males. *p* , significance, Mann–Whitney test is given for differences between females and males.

**Fig. 3:**
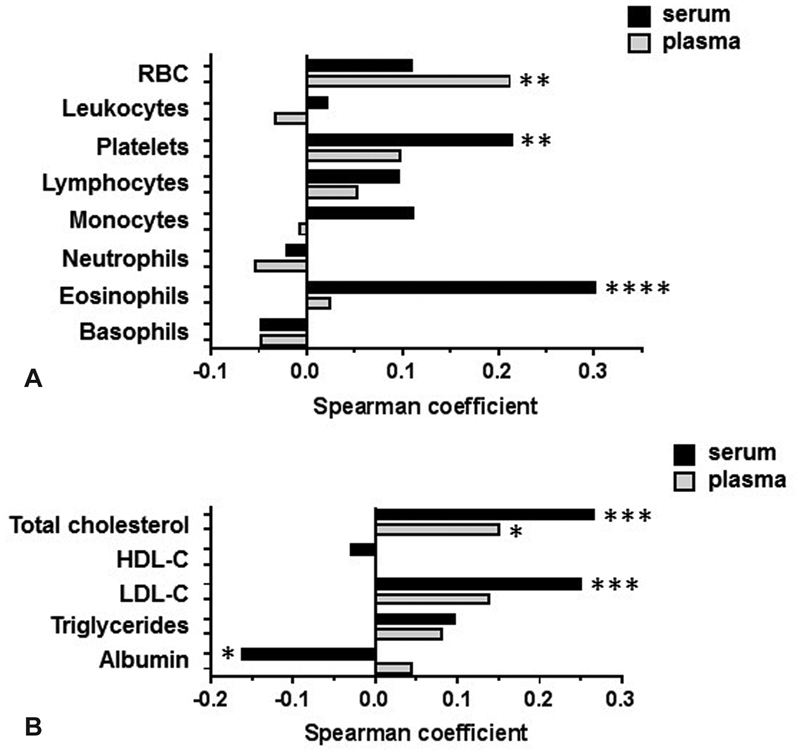
Associations between circulatory S1P concentrations and laboratory parameters. Serum-S1P- ( *black bars* ) and plasma-S1P ( *gray bars* ) concentrations were correlated to cell counts ( **A** ) and lipid fractions ( **B** ) by calculating Spearman coefficients. * *p* < 0.05, ** *p* < 0.01, *** *p* < 0.001, **** *p* < 0.0001; *N* = 173 or 174; nonstarred bars represent statistically insignificant correlations. Note that VLDL-C is assumed to represent a constant fraction of triglycerides (20%). RBC, red blood cell. S1P, sphingosine-1-phosphate; VLDL-C, very low-density lipoprotein-cholesterol.

**Fig. 4:**
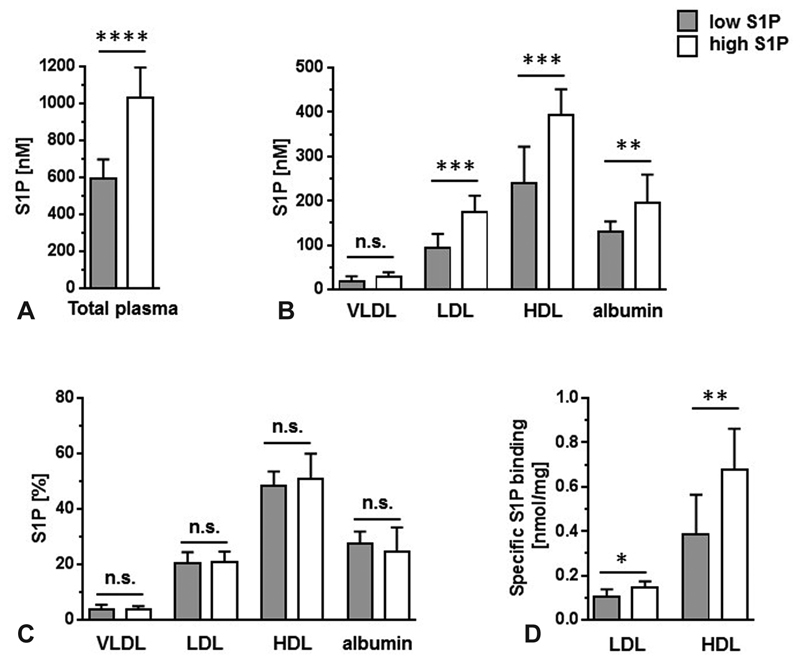
The distribution of S1P in plasma fractions is independent of total plasma-S1P concentrations. ( **A** ) Plasma from 10 study group members each with high ( *white bars* ) or low ( *gray bars* ) plasma-S1P concentrations was fractionated into a VLDL-, LDL, HDL-, and a nonlipoprotein fraction (albumin) and S1P was then measured in each fraction. Data are presented as mean ± SD. ( **B** ), Absolute S1P concentrations are presented. ( **C** ) The relative S1P content in percent is shown for each fraction (100% = VLDL-S1P in % + LDL-S1P in % + HDL-S1P in % + albumin-S1P in %). ( **D** ) Specific S1P binding for LDL and HDL was calculated by dividing LDL-S1P (nM) by LDL (mg/L) and HDL-S1P (nM) by HDL (mg/L), respectively. See Table 1 for HDL and LDL concentrations. Groups were compared by Mann–Whitney test. * *p* < 0.05, ** *p* < 0.01, *** *p* < 0.001, **** *p* < 0.0001. n.s., nonsignificant. HDL, high density lipoprotein; LDL, low density lipoprotein; S1P, sphingosine-1-phosphate; VLDL, very low-density lipoprotein.

To identify potential cellular sources that regulate S1P levels in serum or plasma, correlation analyses between S1P concentrations and counts for RBCs, lymphocytes, monocytes, neutrophils, eosinophils, basophils, and platelets were performed. Serum-S1P concentrations are significantly associated with platelet and eosinophil counts, while for plasma-S1P, the only one significant association found was with RBC counts ( Fig. 3A ). As circulatory S1P is bound to lipoproteins or serum-albumin, correlations between S1P concentrations and total cholesterol, HDL-C, LDL-C, triglycerides (VLDL), and serum-albumin were determined. Notably, it is assumed that VLDL-C is represented by triglyceride measurements based on the common practice to consider VLDL concentrations being a constant 20% of triglyceride concentrations. S1P in both serum and plasma is associated with total cholesterol while no associations were found with triglycerides (VLDL) or HDL-C. An association with LDL-C as well as an inverse association with albumin was observed for serum-S1P only ( Fig. 3B ). To address the question whether the distribution of S1P among lipoprotein fractions is dependent on total plasma S1P concentrations, plasma-S1P fractions were analyzed in 20 subjects, ten of which had high and ten had low total plasma-S1P concentrations ( Fig. 4A ; Table 2 ). The two groups also differed in age, LDL-C concentrations, and platelet counts, all of them are higher in the group with higher plasma S1P levels ( Table 2 ). In contrast, RBC counts and HDL-C concentrations are the same between the two groups ( Table 2 ). VLDL-, LDL-, HDL-, and lipoprotein-free (serum-albumin) fractions were generated by sequential ultracentrifugation and S1P was then measured in each fraction ( Fig. 4B,C ). In all major S1P-containing fractions (LDL, HDL, and albumin) higher S1P concentrations were always measured in the group with higher total plasma-S1P ( Fig. 4B ) and the relative distribution of S1P among the individual fractions remains the same ( Fig. 4C ). The largest fraction is HDL with 48 to 51%, followed by serum-albumin and LDL with 25 to 28% and 20 to 21%, respectively ( Fig. 4C ). The smallest fraction is VLDL that is associated with only 4% of the total plasma-S1P. The larger HDL-S1P fraction in people with high total plasma-S1P is exclusively due to a 1.8-fold increase in specific binding of S1P to HDL ( Fig. 4D ). In contrast, the higher LDL-S1P fraction in these people is mainly due to their increased LDL-C concentration ( Table 2 ; Fig. 4D ).

**Table 2:** Subgroup comparison between subjects with low and high S1P concentrations

		Low plasma-S1P	High plasma-S1P	*p* -Value
Number		10	10	
Age (y)		51.4 (40–57)	56.3 (41–71)	<0.05
Gender (% male)		70	40	n.s.
Serum-S1P	(nmol/mL)	0.72 ± 0.25	1.44 ± 0.34	<0.01
Plasma-S1P	(nmol/mL)	0.59 ± 0.11	1.03 ± 0.17	<0.0001
RBC	(1/pL)	4.73 ± 0.26	5.01 ± 0.54	n.s.
Leukocytes	(1/nL)	5.77 ± 1.56	6.44 ± 1.91	n.s.
Platelets	(1/nL)	197 ± 41	249 ± 21	<0.05
Lymphocytes	(1/nL)	1.46 ± 0.38	1.73 ± 0.36	n.s.
Monocytes	(1/μL)	389 ± 97	484 ± 206	n.s.
Neutrophils	(1/nL)	3.62 ± 1.11	3.90 ± 1.74	n.s.
Eosinophils	(1/μL)	156 ± 59	187 ± 88	n.s.
Basophils	(1/μL)	100 ± 0	100 ± 0	n.s.
Total cholesterol	(mg/dL)	188 ± 37	209 ± 22.4	n.s.
HDL-C	(mg/dL)	67.2 ± 16.9	62.7 ± 23.4	n.s.
LDL-C	(mg/dL)	87.9 ± 24.2	120.1 ± 25.1	<0.05
Triglycerides	(mg/dL)	155 ± 144	131 ± 60	n.s.
Albumin	(g/L)	43.3 ± 2.2	43.8 ± 2.2	n.s.

Abbreviations: HDL-C, high density lipoprotein-cholesterol; LDL-C, low density lipoprotein-cholesterol; n.s., nonsignificant; RBC, red blood cells; S1P, sphingosine-1-phosphate.

Note: Age (mean, min–max), gender, circulatory S1P concentrations (mean ± SD) and clinical laboratory data (mean ± SD) are shown for two groups of 10 study participants each selected for low and high plasma S1P concentrations. *p* , significance, Mann–Whitney test.

## Discussion

The mean serum-S1P concentration measured in this healthy study group and its overall independence of gender ( Table 1 ; Fig. 1 ) are in agreement with a recently published epidemiological study comprising 1,339 individuals. 21 Unexpected may be the finding of higher serum-S1P levels specifically in older women ( Fig. 2 ). In the aforementioned epidemiological study, 21 however, women 60 years and older also showed the trend of higher serum-S1P levels. Gender-specific differences in aging include the immune system and is reflected by differences in lymphocyte populations and expression of inflammatory mediators. 23 It would be worth investigating to what extent elevated S1P levels in older women contribute to these differences.

The rate limiting step in S1P production is the phosphorylation of sphingosine by sphingosine kinase (Sphk1 or Sphk2). Concomitant deletion of both kinases in mice is lethal due to improper neural and vascular development 24 but animals expressing one kinase allele only survive with very low plasma-S1P concentrations. 25 Transplantation of wild-type bone marrow into these animals restores plasma-S1P concentrations to normal levels demonstrating its hematopoietic source. 25 RBCs can readily phosphorylate sphingosine to S1P and store large amounts of S1P as they lack S1P-lyase activity. 26 Upon cell fractionation of human blood, only RBCs were found to be capable of S1P release. 26 Taken together, it was concluded that RBCs are the major source for plasma-S1P. In agreement with this conclusion, RBCs are the only cells found in this study that are significantly associated with plasma-S1P concentrations ( Fig. 3A ). An interesting implication of this work is that major blood antigens may contribute to the wide spread of circulating S1P concentrations in humans ( Supplementary Fig. 1 ). Activated platelets release S1P and thus, serum-S1P concentrations have previously shown to correlate with platelet counts. 17 18 In this study, the difference between serum- and plasma-S1P was 0.3 nmol/mL in average ( Table 1 ; Fig. 1A ) and as predicted, serum-S1P concentrations were found to be associated with platelet numbers ( Fig. 3A ). Surprisingly, the most significant association for serum-S1P, however, was observed with eosinophil counts ( Fig. 3A ). Whether eosinophils directly produce and release S1P, or whether they stimulate the S1P release by other cells in a paracrine manner, remains to be investigated.

In vitro, S1P is extracted from RBC by HDL and serum-albumin, 27 both of which represent the major S1P carriers in plasma. 4 As one may expect, total cholesterol concentrations were found to correlate with plasma-S1P ( Fig. 3B ). This, however, was rather due to correlations to LDL- and VLDL- than to HDL-C ( Fig. 3B ) despite HDL being the major carrier for S1P in plasma. 4 This can be explained by our observation that the specific binding for S1P to HDL is highly variable ( Fig. 4D ). An unexpected observation is the inverse correlation between S1P and albumin in serum ( Fig. 3B ). One possibility is that during coagulation, a novel high affinity S1P carrier is released that extracts S1P from albumin. This scenario is supported by the fact that albumin binds S1P with very low affinity as the dissociation constant for albumin is more than 3 orders of magnitude higher when compared with HDL or LDL. 28 Serum-S1P concentrations correlate with total cholesterol and LDL-C, but not with HDL-C and the same pattern is also observed for plasma-S1P, though with lower correlation-coefficients ( Fig. 3B ). To address the question whether the distribution of circulatory S1P among the various protein carriers depends on total plasma-S1P concentrations, we compared people with high and low plasma-S1P ( Fig. 4 ). Clearly, the S1P content in all major fractions is increased in people with high plasma-S1P ( Fig. 4B ) and if one plots the relative S1P distribution, there is no significant difference between people with low or high total plasma-S1P ( Fig. 4B ). There is a remarkable difference though between LDL and HDL regarding S1P binding. The LDL-S1P fraction increases with higher plasma-S1P mainly because of an increase in LDL-C ( Table 1 ) which means that the specific binding of S1P to LDL only slightly increases ( Fig. 4D ). In contrast, people with higher plasma-S1P do not have more HDL-C ( Table 2 ) and the increase of the HDL-S1P fraction is mainly due to an increase in specific S1P binding ( Fig. 4D ). These observations explain at least in part why for HDL-C concentrations, no significant associations with circulatory S1P concentrations have been observed ( Fig. 3B ).

In conclusion, circulatory S1P concentrations in humans are highly variable and if one considers to utilize S1P as a biomarker or pharmaceutical target it is necessary to know the parameters that define circulatory S1P concentrations. This study identifies eosinophils as a source for serum-S1P and specific S1P-binding to HDL to be variable in apparently healthy blood donors. Limitations of this study include a selective study group of blood donors and therefore, larger epidemiological studies should be performed to confirm these findings.
