# Supplementary material for: Determinants of Serum- and Plasma Sphingosine-1-Phosphate Concentrations in a Healthy Study Group
Source: TH Open. 2020 Jan 23;4(1):e12–9. doi: 10.1055/s-0040-1701205 (PMC6978167; doi:10.1055/s-0040-1701205)
Supplement: Supplementary file 1 — Supplementary Material [file 10-1055-s-0040-1701205-s190047.pdf]

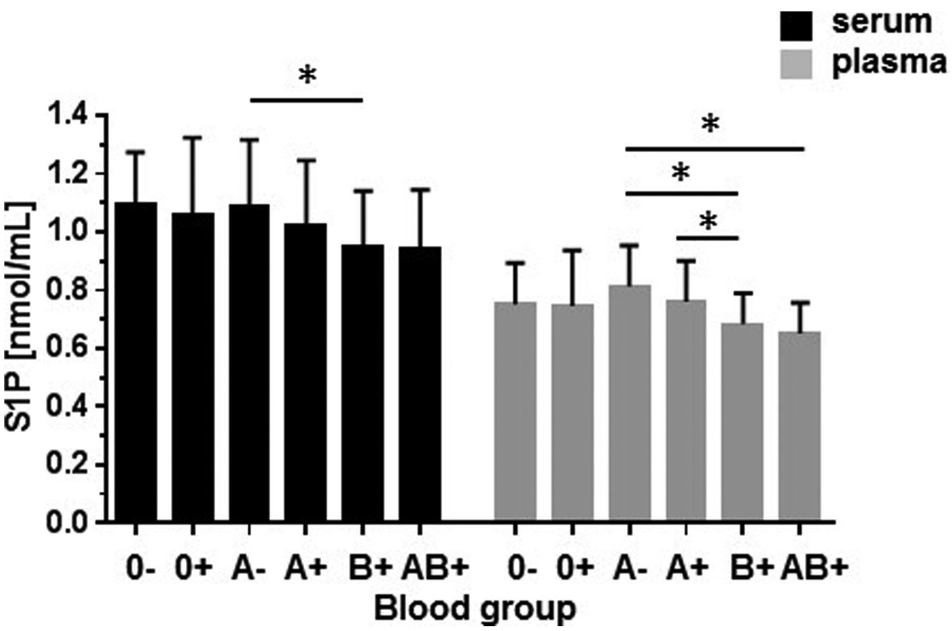

Supplementary Fig. 1 Influence of major blood group antigens on circulatory S1P concentrations. Data (mean ± SD) were stratified by blood groups (±refers to Rhesus factor): 0− ($n = 10$), 0+ ($n = 68$), A− ($n = 12$), A+ ($n = 59$), B+ ($n = 17$), and AB+ ($n = 5$). The blood groups B− ($n = 2$) and AB− ($n = 1$) were not considered. $^{*}p < 0.05$ (Mann–Whitney test). Testing for group differences using the Kruskal–Wallis test revealed no significant differences regarding serum- or plasma-S1P. S1P, sphingosine-1-phosphate; SD, standard deviation.
